# Is nutritional functional diversity in the rural food and nutrition system associated with food security and nutrient adequacy? A case study of rural areas of Zahedan district, Iran

**DOI:** 10.1186/s12889-022-13134-8

**Published:** 2022-04-14

**Authors:** Mahdieh Sheikhi, Nasrin Omidvar, Seyed Mehdi Tabatabaei, Hassan Eini-Zinab

**Affiliations:** 1 Department of Community Nutrition, National Nutrition and Food Technology Research Institute; and Faculty of Nutrition Sciences and Food Technology, Shahid Beheshti University of Medical Sciences, No. 7, Hafezi St., Farahzadi Blvd, Tehran, Iran; 2 Health Promotion Research Center, Zahedan University of Medical Sciences, Zahedan, Iran

**Keywords:** Nutritional functional diversity, Food system, Food security, Nutrient adequacy, Rural, Iran

## Abstract

**Background:**

An overlooked problem in food and nutrition system analysis is assuring adequate diversity for a healthy diet. Little is known about nutrient diversity in food and nutrition systems and how it transmits to dietary diversity. Nutritional functional diversity (NFD) is a metric that describes diversity in providing nutrients from farm to market and the consumption level. The objective of this study is to determine the NFD score at different stages of the rural food and nutrition system, including household’s agricultural and home production, domestic food processing, purchased food, and diet. It also aims to explore the association between NFD and nutrient adequacy, food security, and anthropometric indicators.

**Methods:**

A cross-sectional study was conducted on 321 households in 6 villages of Zahedan district. The NFD score was measured at three subsystems (production, processing, and consumption) of the food and nutrition system. Household food security, mean adequacy ratio (MAR), and anthropometrics of the household’s head were measured to assess the association between NFD and food and nutrition indicators. Linear and bivariate statistical techniques were applied to study the associations between variables.

**Results:**

In the rural food and nutrition system, the food purchased from the city plays the main role in the households NFD score. Their contribution to total NFD was twice that of the food items purchased from the village. The NFD score of homestead production and households food processing was found to be five times less than those of food purchased from cities. The food insecure households had significantly lower NFD scores for food purchased from the city and higher NFD scores for purchased food items from the rural market and native wild vegetable consumption. A strong and positive relationship was observed between NFD of food items purchased from the city and households’MAR. No significant association was found between the NFD score of homestead production, processing, and dependent variables, i.e. food insecurity, MAR, and household head anthropometrics.

**Conclusion:**

NFD score, as a relatively new metric, could help in determining diversity from farm to diet and identifying the gaps to plan appropriate interventions for improving diversity in the local food system.

**Supplementary Information:**

The online version contains supplementary material available at 10.1186/s12889-022-13134-8.

## Background

The trend of hunger, after decades of steady decline, has been slowly on the rise worldwide since 2014 [1]. More than 768 million people all over the world are still hungry and 2.3 billion people have no regular access to safe, sufficient, and nutritious food, and are suffering from moderate to severe levels of food insecurity [2]. Given these conditions, the goal of achieving zero hunger by 2030 seems unrealistic [3, 4]. COVID-19 pandemic, disproportionate slowdowns or downturn economy, climate variability, and conflicts are currently exacerbating these trends [3, 5].

In recent decades, the global burden of chronic hunger (calorie deficiencies) has declined much more rapidly than the global burden of hidden hunger (micronutrient deficiencies) [6]. While receiving sufficient calories is still considered a major challenge, an overlooked problem in food and nutrition systems is having access to adequate diversity of nutrients for providing a healthy diet and life [7]. It is well recognized that hidden hunger and malnutrition have roots in dysfunctional food and nutrition systems; the integrated model developed by *Sobal *et al*.* included three subsystems (production, consumption, nutrition) [8], that is accompanied by nutrient inadequacy of diet, especially in poor communities [9–12].

Diversity throughout the food and nutrition system approach could be considered as a driver for change by improving direct interactions between food producers and consumers, increasing the quality of diet, and fighting the triple burden of malnutrition (undernourishment, micronutrient deficiencies, and overweight and obesity). Such an approach can also be associated with lower rates of food insecurity and mortality [13–20].

One of the main gaps in food diversity research is that they often tend to deal with food diversity in one or two subsystems, and as a result, a holistic perspective that encompasses food diversity in all subsystems of the food and nutrition system is missing [21, 22]. Various indicators have been used to assess food diversity but the focus has been mainly on consumption rather than food production and provision. Little is known about how diversity in production and provision is transmitted to dietary diversity at the household level [23]. For this purpose, proper metrics are required to assess the ongoing food policy at both national and regional levels of the food and nutrition system to modify or design appropriate interventions for its improvement.

Nutritional Functional Diversity (NFD) is a metric first introduced by *Remans *et al*.* [24], to describe diversity in available nutrients from farms, markets, and the consumption level. NFD shows nutritional differences and variations in all groups of foods and food items that are not captured by a food variety score and/or a diet diversity score [25]. Notably, the NFD score can be used at any level (from farm to diet), because it is based on the nutritional composition of food for 17 nutrients that play key roles in human health [24]. Therefore, the NFD score can reflect the potential of a food system in meeting the nutritional requirements of the population as well as the link between subsystem diversity, food security, and health. This issue has not been well-addressed in the studies conducted on the food and nutrition systems in Iran.

This study aimed to determine the NFD score in different subsystems of rural food and nutrition system from households agricultural and homestead production and processing, food purchased, and diet. Moreover, this research aims to explore the association of the NFD score of rural food and nutrition system with nutrient adequacy, food security, and anthropometrics in rural communities of Zahedan. Zahedan is the center of Sistan and Baluchistan province located in Southeast, Iran. The province has the highest food insecurity and hidden hunger rate in the country where more than 70 percent of the population live in rural areas [26, 27]. Identifying the rural food and nutrition system diversity dynamics can help policymakers to design targeted interventions to fight against malnutrition.

### Conceptual framework

The Nutritional Functional Diversity indicator is a new concept in food and nutrition research and data on its linkage to food and nutrition security and the related dimensions are still scarce [23, 24]. NFD indicator can be applied to describe the diversity of food at different stages of the food and nutrition system. Little is known about food diversity at different stages of rural food and nutrition systems and the way it transmits to dietary diversity at the household level [23, 28]. *Remans *et al*.* developed NFD to address nutritional diversity in farming systems and the link between NFD, food security, and nutrition indicators, including anthropometry and nutrient deficiencies. They suggested establishing linkages between nutritional diversity and consumption and human health outcomes as a major strategy for research. They also proposed potential determinants of NFD, including agro-ecological, socio-economic and cultural factors, and seasonality of products at both farm and village levels [24]. Based on another study by *Bellon *et al., there is a triangular connection among three facets of diversity on-farm, market, and diet [29]. Moreover, endogenous connections exist between on-farm diversity and dietary diversity through self-consumption, between on-farm diversity and market diversity through sale, and between dietary diversity and market diversity through purchase. These are all affected by some confounding factors such as land quality and tenure, climatic variability, different types of markets, and ethnicity [29].

**Fig. 1:**
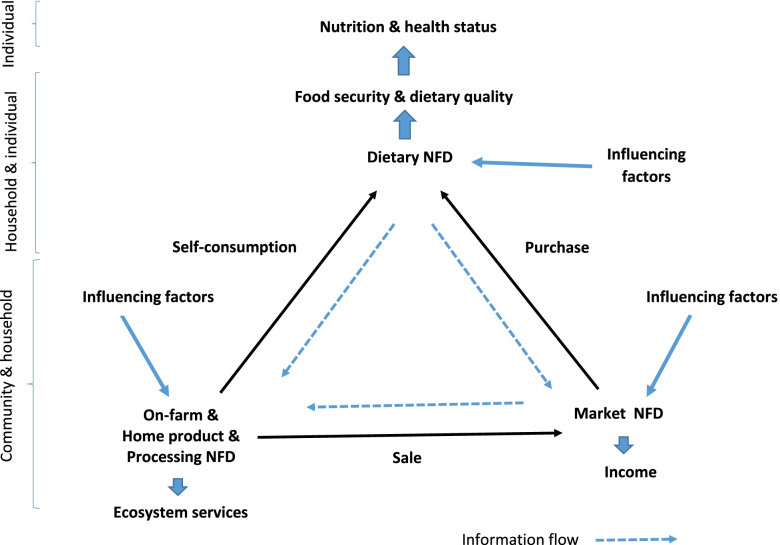
Conceptual framework on the linkage among the NFDs at the farm, homestead production, household’s processing, purchased food, and diet in rural food and nutrition system [29]

The conceptual framework of this study combines the two above-mentioned concepts (see Fig. 1). The NFD score is measured in the food and nutrition system of rural households similar to the conceptual model of Bellon et al., which provides a holistic framework of the relationships between on-farm, market, and dietary diversity. Correspondingly, the food and nutrition system of rural households is a local system that gathers all the elements and activities related to the production, processing, distribution, preparation, and consumption of food, as well as the outputs of these activities, including food security, socio-economic, and environmental outcomes [30]. This study paid specific attention to NFD score in rural food and nutrition system and its association with nutrition and health outcomes. We hypothesized that in rural food and nutrition system, NFD on the farm, homestead production, and households processing are linked through the following two routes: on market through the sale and dietary diversity through self-consumption. Agroecology, including climatic, soil types and conditions, water quality, greenhouse gas emissions, native species diversity and distribution of species, socio-economic condition (including access to a diversity of seeds, fertilizers, knowledge, and market(s)), and socio-cultural factors (including cultural preferences for species and subspecies, and multiple purposes and advantages of crops) can affect NFD on-farm and home products. Homestead production of diverse types can play an important role in providing enhanced food supply and increased dietary diversity. Various studies have previously shown that combining home garden and poultry production could improve food insecurity, malnutrition, and anemia among children and women [31–33].

Market NFD was indicated to be linked with dietary NFD through purchase (from a city, village or as a gift, etc.), which is influenced by a set of factors, e.g., market access and place, links to different types of markets, availability of infrastructure, food price, households’ budget, ethnicity, and knowledge. In this regard, market linkages would enable households to consume diverse diets by enhancing household's ability to sell part of their produce to gain extra income, which in turn will increase household's ability to access more diverse and nutritious food, especially during the lean season [28, 34]. In rural areas of the developing countries worldwide, markets often have poor functions. Rural markets are highly variable in what they provide, and access to urban markets is often difficult for low-income households due to a lack of road infrastructure and proper transportation [35–37].

The the NFD score in rural food and nutrition system affects food and nutrition indicators, including food and nutrition security, dietary quality, anthropometrics, and nutrient deficiencies, which are in turn affected by factors such as family size, age, sex, and educational level of households head, household’s income and welfare, and distance from the market. The relationship between NFD scores at different subsystems of rural food and nutrition system with each other and with other food and nutrition indicators does not seem to be a simple linear association. Accordingly, this relationship can be complex, depending on different regions and various factors.

## Methods

### Setting and study design

This population-based cross-sectional study was conducted in Zahedan rural communities from April to July 2019. Zahedan is the capital city of Sistan & Baluchistan province with long, sweltering, arid, and clear summers and cold, dry, and mostly clear winters; with a minimal amount of rain throughout the year [26]. Based on the "food security information and mapping system in Iran" survey [27], this province has the highest food insecurity rate in the country (58.8 percent) [38], where 12.8% and 20.7% of children aged under five years old are suffering from underweight and stunting, respectively [27]. Zahedan is subdivided into three administrative areas: Central, Kurin, and Nosratabad. Two villages from each area were randomly selected. According to Cochran's formula and based on the rural household’s population, the sample size was calculated as 220 household for a 95% confidence level. In two villages with less than 60 households, a census was conducted. To get an overview of the state of the rural food and nutrition system, the sample size was increased to 321 households.

### Data collection

Before starting the fieldwork, 12 interviewers who were fluent in the local language of the region, i.e., Baluchi, were recruited from community health staff members. Thereafter, they were trained in a two-day workshop to improve coordination and reduce the chances of interpersonal variation in the data collection process. The required data were collected through face-to-face interviews with mothers (assisted by their husbands, if they were present).

### Measurements

#### Household’s demographics

Demographic information and socioeconomic variables, including age, gender, educational level, employment of all household members, household size, household subsidy support, and household facilities were collected using a validated questionnaire by Statistical Center of Iran [39]. To calculate households welfare index, the household’s amenities and belongings were recorded and weighed based on price, necessity, and importance between 0–10 by the research team. The total welfare score was computed by adding weighted scores. The final categorical variable (i.e. low, medium, and high welfare groups) was created using tertile cut points.

#### Household’s agricultural and homestead production, and processing

Households were asked to report details of their agricultural and home production and processing during the last 12 months using a questionnaire consisting of the following 3 sections: 1) existence of home-garden and household’s processing operations performed on raw foodstuff to increase their shelf life, as well as the type and amount of produced/processed product(s). Examples of household food processing include making jam, yogurt, and drying fruit and vegetables. 2) Presence of livestock/poultry in the household, its type and quantity, and 3) ownership of agricultural land, type of crop(s) cultivated, and the amount produced.

#### Household’s dietary intakes and food sources

Household’s dietary intakes and access to different types of markets were recorded by trained community health workers by completing 24-h diet recalls for two non-consecutive days in a week. All foods and dishes consumed by the households and their ingredients were recorded according to the main meals and snacks and also according to the source of each food including purchased from any place, own production, and/or gift, and native wild vegetables (edible wild vegetation that grows naturally around the villages).

The interviewee was asked to bring the same amount of ingredients used to prepare food and the interviewers weighed all items using a calibrated digital scale with a precision of ± 1 g. (Kitchen Scale EK8450, Camry Electronic Ltd, Guangdong, China). If food items were not available, the intake values were converted to grams, based on household food scales [40], then the percentage of non-edible food were deducted and the raw to cooked coefficients were applied [40].

Nutritional values of the consumed food items were calculated using the Iranian food composition table [40] and the USDA Food Data Central [41]. Considering the dietary reference intake (DRI) for energy, protein, and limiting micronutrients in the region, including vitamin A, calcium, iron, and zinc [42, 43], the adequacy of household’s dietary intake was estimated by calculating household's adult male equivalent units (AMEs) using the method introduced by Weisell et al*.* [44]. AMEs were computed according to the energy requirement of an adult male aged 18 to 30 years old with a moderate level of physical activity [45, 46]. Considering a number of the present household members and guest(s) per meal, their age, sex, the AME for each meal was calculated separately. Daily meals, including breakfast, lunch, dinner, and snack(s) were calculated with relative weights of 0.16, 0.43, 0.30, and 0.11, for each meal, respectively. This weighting value was then applied to each meal representing the energy contribution of that meal to the daily energy intake, which is an important factor to be considered when the food consumed throughout the day is measured which is often neglected [44]. Finally, the adequacy of the household’s dietary intakes was assessed by dividing the household’s dietary intakes for energy and micronutrient by household’s AME. Nutrient adequacy ratio (NAR) was calculated for each nutrient as the percentage of the nutrient meeting the DRI for energy, protein, and 10 other micronutrients, including iron, zinc, calcium, vitamins A, C, B1, B2, B3, B12, and folate. The mean adequacy ratio (MAR) of the diet was calculated by summing the 12 NARs and dividing by the number of nutrients [47].

#### Nutritional Functional Diversity (NFD) score

NFD is based on the functional differences of available foods in a food and nutrition system and higher functional scores indicate a more diverse diet. The NFD score of the food and nutrition system was measured at three levels: 1) in the production subsystem via a researcher-made questionnaire by measuring various annual households agricultural and home products, 2) in the processing subsystem using a questionnaire by measuring various annual household’s processing levels, and 3) in consumption subsystem by obtaining household’s 24-h diet recalls for two non-consecutive days, which also included some questions on place of purchasing/obtaining the food items and their prices.

In this study, the NFD score was calculated according to the four main steps described by Luckett et al*.* [23]: firstly, a food–nutrient matrix was created. In this matrix, each row contained one of the food items in the rural food system, and each column contained a nutrient, so each cell of the matrix gave the nutrient content of food items. In all levels of the rural food and nutrition system, after excluding foods with negligible nutritional value (e.g., salt and spices), 133 food items were selected to be included in the final analysis. In this regard, the food–nutrient matrix was composed of energy and the following sixteen nutrients: protein, fat, carbohydrate, fiber, vitamin A, vitamin D, vitamin C, thiamin, riboflavin, niacin, folate, vitamin B12, Ca, K, Fe, and Zn. Nutritional values were calculated per 100 g of the food items obtained from the Iranian food composition table [40] and the USDA Food Data Central [41]. Next, the nutrient values in the food matrix were standardized in the following two ways: 1) they were divided by the recommended dietary allowances (RDA) for adult males aged between 18 and 30 years old, and 2) results were then standardized to have mean = 0 and SD = 1. Thirdly, the food–nutrient matrix was converted into a food–food distance matrix. Finally, the distance matrix was used to produce a cluster diagram, called a dendrogram, which was used to calculate the NFD score.

#### Household food security

Household’s food security status was assessed by the household food insecurity access scale (HFIAS), which has been validated for the Iranian population [48]. This questionnaire consists of nine Likert-type questions on a four-week recall period. The respondent was first asked an occurrence question, as to whether the condition in the question happened in the past four weeks (yes or no). If the response was “yes” to the occurrence question, frequency-of-occurrence was then asked to determine whether the condition happened rarely (once or twice), sometimes (three to ten times), or often (more than ten times) for him/her in the past four weeks. The HFIAS categorizes households into the following four levels of food insecurity levels: food secure, mild, moderate, or severe food insecure [49].

#### Anthropometrics

Body weight and height of the household’s head and his spouse were measured using a digital scale (Seca, Germany) and body mass index (BMI) was then calculated as weight (kg)/height (m^2^). Weight status of those aged between 19 and 60 years old was classified into one of the following classes: underweight (BMI < 18.49), normal weight (18.5–24.99), overweight (25–29.99), and Obese (> 30 kg/m^2^) [50, 51]. In older individuals (≥ 60 years old), BMI cut-offs were defined as: ≤ 20.9 (wasting), 21–26.9 (normal), 27–29.9 (overweight), and ≥ 30 (obese).

Waist circumference was also measured with a precision of 1 mm in young and middle-aged individuals with a non-elastic tape at mid-way between the lowest rib and the iliac crest. Abdominal obesity was defined as waist circumference of more than 80 cm in women and 94 cm in men [52].

### Statistical analysis

Data were expressed as mean ± standard error (SE) or percentages. The Kolmogorov–Smirnov test and histograms were used to test the normality of the interval/ratio scale variables. After confirming the non-normal distribution of the quantitative variables, nonparametric Mann–Whitney and Chi-square tests were both performed to compare the outcomes between food secure and insecure households. The student’s T-test was used if the data distribution were normal.

The NFD scores were calculated using the R software (version 4.0.4). The associations between NFD scores at different levels of food and nutrition system with households food security were analyzed using binary logistic regression. The associations between NFD scores with micronutrient adequacy, BMI, and waist circumference of household’s heads were analyzed using linear regression. Odds ratios (OR) and regression coefficients with 95% confidence intervals (CI) were calculated both with and without control variables in regression models.

For the food security model, the control variables included family size, household’s income, households having additional subsidy plan (except for national subsidy), distance from the city, household head’s sex, educational level, and employment, and household’s welfare index. The control variables for the mean adequacy ratio model included the same variables as the food security model, but instead of sex, the age of the household's head was used. For the BMI of the household’s head model, control variables of the mean adequacy ratio model plus residence status were included as control variables. The For waist circumference of the household’s head model, control variables were family size, age, sex, and marital status of the household’s head, household’s income, household being covered by an additional subsidy plan (except for national subsidy); and household’s welfare index. All the without control models were run with no control variables.

Data analysis was performed using Statistical Package for the Social Sciences (SPSS) version 16.0 (SPSS Inc., Chicago, IL, USA) software. A *p*-value of 0.05 or less was considered the statistically significant level.

## Results

### Household’s demographic characteristics

Data were obtained from 324 households selected from 6 villages (an average of 54 households per village). The average family size was 4.6 and 30% of the households had more than six members. About 70% of the household heads were middle-aged, 82% were men, more than 60% illiterate or with primary school educational level, and 40% were unemployed/housewife (Table 1). In Iran, households with income levels under 70 percentile of income distribution [53] are covered by the national unconditional cash transfer (UCT) program. This program covered 98.8% of the households studied and 23.5% received additional help through Imam Khomeini Relief Fund.

**Table 1:** Characteristics of households included in the study, Zahedan rural areas

Variables	Household’s food security status			
	Total household	FS^1^	FI^2^	
	(*n* = 321)	150 (46.7%)	171 (53.3%)	
	Mean (SE)	Mean (SE)	Mean (SE)	*p*-value^†^
Family size (persons)	4.64 (0.11)	4.28 (0.15)	4.98 (0.16)	0.004
Age of household head (years)	43.57 (0.82)	43.72 (1.39)	43.45 (0.95)	0.235
Household income (Rial^3^)	10,309,304.6 (573,032.7)	13,361,259.0 (994,321.5)	7,644,593.8 (565,466.2)	< 0.001
Household’s income excluding subsidy (rial^3^)	7,783,696.4 (564,935.8)	10,957,500.0 (982,080.1)	5,008,812.5 (540,812.1)	< 0.001
Village distance from the city (Km)	68.90 (1.27)	64.76 (1.65)	72.77 (1.88)	0.006
MAR^4^	67.43 (0.75)	69.50 (1.11)	65.74 (1.01)	0.009
NFD^5^ household’s agriculture, (*n* = 17 households) (5.2%)	1.4 (0.5)	-	-	-
NFD of homestead production (*n* = 145 households) (44.8%)	3.72 (0.20)	3.5 (0.34)	3.83 (0.26)	0.415
NFD of household’s food processing, (*n* = 141 households) (43.5%)	3.69 (0.25)	3.57 (0.36)	3.77 (0.35)	0.966
NFD of Consumption				
Food purchased from the city, (*n* = 311 households) (96%)	15.92 (0.34)	17.58 (0.47)	14.53 (0.48)	< 0.001
Food purchased from village, (*n* = 277 households) (85.5)	6.93 (0.37)	6.05 (0.53)	7.72 (0.51)	0.021
Received gift, (*n* = 159 household’s) (50.9%)	3.24 (0.32)	2.65 (0.41)	3.62 (0.48)	0.374
Native wild vegetables, (*n* = 208 households) (64.2%)	1.23 (0.08)	0.98 (0.12)	1.40 (0.12)	0.029
Household’s dietary, (*n* = 321 Households) (100%)	16.28 (0.19)	16.62 (0.31)	16.00 (0.25)	0.280
	n (%)	n (%)	n (%)	*p*-value^††^
Household’s Welfare index				
Low	106 (32.7)	42 (40)	63 (60)	0.002
Medium	107 (32.7)	41 (39)	64 (61)	
High	111 (34.3)	66 (60)	44 (40)	
Residence status				
Ownership	249 (77.7)	117 (47)	132 (53)	0.775
Rent/other	71 (22.3)	32 (45.1)	39 (54.9)	
Households received additional subsidies (in addition to the national subsidy)				
Yes	79 (23.5)	32 (42.7)	43 (57.3)	0.421
No	248 (76.5)	118 (48)	128 (52)	
Household head characteristics				
Gender				
Male	266 (82.1)	128 (48.5)	136 (51.5)	0.175
Female	58 (17.9)	22 (38.6)	35 (61.4)	
Education level				
Illiterate and primary	215 (67)	94 (43.7)	121 (56.3)	0.124
High school and higher education	106 (33)	56 (52.8)	50 (47.2)	
Employment				
Employed	188 (58.9)	104 (55)	85 (45)	0.001
Unemployed/housewife	132 (41.1)	46 (35.4)	84 (64.6)	
Married status				
Married	258 (80.2)	123 (47.7)	135 (52.3)	0.492
Other (Single/Divorced/Widowed)	63 (19.8)	27 (42.9)	36 (57.1)	
Weight status (based on BMI^6^)				
Normal	162 (52.6)	78 (48.8)	82 (51.2)	0.096
Wasted	50 (16.2)	18 (33.3)	36 (66.7)	
Overweight/obese	96 (31.2)	46 (50.5)	45 (49.5)	
Waist circumference				
Normal	236 (77.1)	107 (45.7)	127 (54.3)	0.759
Abdominal obesity	70 (22.9)	33 (47.8)	36 (52.2)	

^1^Food Secure, ^2^Food Insecure, ^3^Rial is the currency of Iran, ^4^Mean Adequacy Ratio, ^5^Nutritional Functional Diversity, ^6^Body Mass Index.^†^ Using Mann–Whitney U test, ^††^Using Chi-squared test

Based on body mass index, about 52% of the household heads had normal weight, 16% were underweight, and 31% were overweight or obese. Abdominal obesity was observed in 22% of the household heads.

### NFD score of rural food and nutrition system

Table 1, shows the results of the NFD scores at different levels of rural food and nutrition system, including household's agriculture, home production, purchased food, and diet. The NFD score for each household at different levels was calculated as a percentage of the total NFD score in each village’s food and nutrition system. The NFD score ranges from 0 to 100 and higher scores indicate greater nutrient diversity.

Most of the households were not farmers and only 5.2% of the households (*n* = 17) had agricultural products with a mean NFD score of 1.4. Approximately, 44% of households were involved in homestead production and mostly produced animal proteins (chicken and goat), dairy products, fruits (pomegranate, mulberry, and grapes), and rarely green vegetables. 23.4% of households sold some of their productions to other rural households. Canned and dry goods imported from the city were often sold in village markets but no local products were sold there.

Purchased foods contributed more to the household’s nutritional diversity than home products. A mean NFD score of 15.92% for food purchased from the city means the contribution of urban markets to households nutritional diversity is 15.92 percent. The NFD contribution of food purchased from the city was twice that of food purchased from the village. About 50.9% of the household’s received food as a gift; however, this had a low contribution to the NFD score. Overall, lower NFD scores at all levels consequently led to low levels of NFD scores in the household diet. Moreover, households with moderate and severe levels of food insecurity had significantly lower NFD scores for the food purchased from the city and higher NFD scores for food purchased from the rural market, as well as native wild vegetable consumption.

### Household’s food security status

The prevalence rate of mild and moderate/severe household food insecurity was 12.7 and 53.3%, respectively. Mild food insecure households constituted a small proportion of the sample and their socio-economic characteristics, i.e., household income and household head’s employment status, were similar to those of the food secure households. Therefore, the two groups were merged into a single group. The proportion of household food insecurity was found to be significantly higher in low income, low welfare index, low MAR, and crowded households. It was also higher for households with unemployed heads and those far from urban areas. No significant difference was observed in anthropometric indices based on household food security status.

### NFD and food security

No significant association was found between NFD scores of home food production, household food processing, and household food insecurity before and after controlling for covariates (including household size, household income, household under additional subsidy plan (except for the national subsidy), distance from the city, head of households sex, educational level and employment, and households welfare index (Table 2).

**Table 2:** Association between NFD score of rural food and nutrition system with households food security, Zahedan rural areas

Predictors	Household’s food security status	
	Without control variables	With control variables^a^
	FI^1^	FI
	OR (CI95%)	OR (CI95%)
NFD of homestead production	1.047 (0.918–1.193)	1.123 (0.930–1.355)
NFD of household’s food processing	1.023 (0.916–1.143)	1.047 (0.899–1.219)
NFD of Consumption		
Food purchased from city	0.916 (0.880–0.955)***	0.928 (0.881–0.978)**
Food purchased from the village	1.045 (1.005–1.088)*	1.051 (1.000–1.104)*
Received gift	1.061 (0.979–1.150)	1.057 (0.961–1.164)
Native wild vegetables	1.311 (1.036–1.659)*	1.195 (0.890–1.604)
Household’s diet	0.952 (0.894–1.013)	0.969 (0.895–1.048)

^1^Food Insecure, Food secure is reference. *p < 0.05, **p < 0.01, ***p < 0.001

^a^ Controlled for family size, income, households received additional subsidy (in addition to the national subsidy), distance from the city, sex and education of household’s head, household’s welfare index, employment of household’s head

NFD score of food purchased from the city was 8.4% lower in food insecure households (it reduced to 7.2% after controlling for covariates). The NFD score of food purchased from the village was 4.5% higher in food insecure households. After controlling for covariates, this difference became even wider (5.1%). Notably, with a one unit increase in the NFD score of native wild vegetables, the odds of being food insecure increased by 31.1%. However, this relationship was not significant once the covariates were controlled (Table 2).

### NFD and mean adequacy ratio

There was an inverse, but statistically not significant, the association between NFD scores of home production, household food processing, and household MAR. A positive and strong association was also found between the NFD score of food purchased from the city and household MAR, which was retained after controlling for covariates (Table 3).

**Table 3:** Association between NFD score of rural food and nutrition system with household’s Mean Adequacy Ratio, Zahedan rural areas

Predictors	Household’s MAR^1^	
	Without control variables	With control variables†
	B (CI95%)	B (CI95%)
NFD of homestead production	-0.035 (-1.065–694)	0.412 (-0.450–1.275)
NFD of household’s food processing	0.121 (-0.194–1.219)	0.419 (-0.297–1.134)
NFD of Consumption		
Food purchased from city	0.233 (0.273–0.756)***	0.353 (0.108–0.599)**
Food purchased from village	0.032 (-0.192–0.332)	-0.017 (-0.266–0.232)
Received gift	0.146 (-0.036–1.007)	0.458 (-0.059–0.976)
Native wild vegetables	0.127 (-0.100–2.733)	0.876 (-0.504–2.256)

^1^Mean Adequacy Ratio, *p < 0.05, **p < 0.01, ***p < 0.001

^†^Controlled for family size, age of household’s head, household’s income, households received additional subsidy (in addition to the national subsidy), education of household’s head, household’s welfare index, employment of household’s head

### NFD and anthropometric indicators

There was an inverse, but statistically not significant, the association between NFD scores of home production and food processing and BMI and waist circumference of household head (Tables 4, 5). A significant association was found between the NFD score of food purchased from the city and the abdominal obesity of the household head. The odds of being in the abdominally obese category increase by 5.8% with a one unit increase in NFD. This effect faded out after controlling for covariates. Notably, no significant association was found between the NFD score of rural food and nutrition system and the BMI of household head (Tables 4, 5).

**Table 4:** Association between NFD score of rural food and nutrition system with BMI of household head, Zahedan rural areas

Predictors	BMI^1^ of household head	
	Without control variables	With control variables^a^
	B (CI 95%)	B (CI 95%)
NFD of homestead production	0.233 (-0.098–0.563)	0.069 (-0.298–0.435)
NFD of household’s food processing	0.044 (-0.205–0.294)	0.033 (-0.238–0.304)
NFD of Consumption		
Food purchased from city	0163 (0.077–0.249)^***^	0.087 (-0.006–0.180)
Food purchased from the village	-0.035 (-0.125–0.056)	-0.034 (-0.125–0.057)
Received gift	0.20 (-0.173–0.213)	0.058 (-0.122–0.238)
Native wild vegetables	0.218 (-0.294–0.730)	0.078 (-0.468–0.624)
Household’s diet	0.184 (0.041–0.328)^*^	0.033 (-0.124–0.191)

^1^Body Mass Index, *p < 0.05, **p < 0.01, ***p < 0.001

^a^Controlled for family size, age of household’s head, household’s income, households received additional subsidy (in addition to the national subsidy), residence status, education of household’s head, household’s welfare index, employment of household’s head

**Table 5:** Association between NFD score of rural food and nutrition system with waist circumference of household head, Zahedan rural areas

Predictors	Waist circumference of household head	
	Without control variables	With control variables^a^
	B (CI95%)	B (CI95%)
NFD of homestead production	0.608 (-0.262–1.478)	0.147 (-0.803–1.098)
NFD of household’s food processing	0.237 (-0.454–0.928)	0.063 (-0.650–0.777)
NFD of Consumption		
Food purchased from city	0.433 (0.209–0.657)^***^	0.163 (-0.072–0.398)
Food purchased from the village	-0.122(-0.363–0.119)	-0.105 (-0.335–0.124)
Received gift	0.160 (-0.345–0.666)	0.345 (-0.136–0.827)
Native wild vegetables	0.885 (-0.416–2.186)	0.217 (-1.113–1.546)
Household’s diet	0.607 (0.232–0.981)	0.130 (-0.268–0.528)

^*^p < 0.05, **p < 0.01, ***p < 0.001

^a^Controlled for family size, age, and sex of household’s head, household’s income, households received additional subsidy (in addition to the national subsidy), household’s welfare index, the married status of household’s head

## Discussion

This is the first study that has employed the NFD tool at different stages of the rural food and nutrition system in Iran**.** The results show that in the rural food and nutrition system, foods purchased from the city played the main role in the household’s NFD score. In other words, rural households dietary diversity highly relies on the city. Additionally, the NFD score of food purchased from the city was lower in food insecure households and those households farther from the city. Similar findings from a study performed in rural areas of Malawi showed that purchased food contributes more to household nutritional diversity items and households living farther from roads and population centers had lower overall diversity [23]. It was reported that smallholder farming households of Malawi, often purchased more than half of their food from the market [22].

Data on NFD scores and studies on the relationship between farm production diversity and diet diversity are scarce [24, 25, 54, 55]. Also, limited data and information are available on NFD on the continuum of a food system or the association between NFD scores at different food sub-systems and nutrient adequacy and household food security. Moreover, the results of a meta-analysis showed that the effects of production diversity on dietary diversity and nutrition in smallholder farm households are positive, but small [56]. This finding may be due to the role of markets in diet diversity [23, 57]. In the present study, very few households owned agricultural land and the NFD score of crop production was very small. In contrast, more than 40% of the households had homestead production and processing. No association was found between the NFD score of home production/processing, which was low, and the nutritional outcomes, i.e. household food security status, MAR, and anthropometrics. *Remans *et al. in their study in rural areas of Africa did not observe a significant association between NFD of edible crops, household dietary diversity score, and household food security [24].

We found no study examining the role of NFD scores of household food purchases, and diet with food insecurity and adequacy of micronutrients. Food insecure households had a lower NFD score for foods purchased from the city than the NFD score for food purchased from the village and native wild vegetable consumption. Also, the NFD score for diet was low. It seems that the socio-economic factors, such as illiteracy and unemployment of household heads, low levels of welfare index, household size, and village’s distance from the city contribute to low NFD score, nutrient inadequacy, and food insecurity. *Luckett *et al*.* in their research found that some factors such as each additional person in a household, lack of a primary-school education, and household head being older than 50 increase the likelihood of household falling into the lowest quintile of total NFD [23].

One of our intentions in assessing NFD score in rural food and nutrition systems was to identify the relative roles of homestead production, processing, markets, and diet in nutritional diversity as well as to investigate its effect on household nutrient adequacy and food security. Since NFD scores of homestead production and processing were similar for both food secure and insecure households, women empowerment strategies [57–59] can be considered effective approaches both for increasing food diversity in households and reducing food insecurity. In addition, the development of local food markets with more affordable prices can increase households’ access to diverse food items [21, 29]. Finally, although the NFD score of native wild vegetables was low, it was indicated that they can play an important role in the household diet in certain seasons, especially in food insecure households.

### Strengths and limitations

One of the strengths of this study is that the food and nutrition system was seen at different stages, while most studies in this field have mainly focused on the relationship between production diversities or diet diversity and nutritional consequences [25, 56]. Furthermore, examining the relationship between NFD score and various nutritional outcomes, i.e., food security status, adequacy of micronutrients, and anthropometrics was another important point of the current study, while previous studies have often examined one or two nutritional outcomes.

However, the results of this study should be interpreted with some caution due to some limitations. It should be noted that we were not able to assess some factors affecting the NFD score of home products, including soil and water quality, garden size, and environmental factors. Half of the surveyed villages had no access to piped water and their sources of agricultural water were saline water wells. Therefore, improving water access for subsistence farms seems to be a more promising development strategy.

The NFD of purchased food was calculated from the data by two non-consecutive 24 h dietary recalls, which did not include any seasonal difference. Analyzing the NFD in household diets and food sources in different seasons would require data collection at different seasons.

## Conclusion

A holistic perspective on food diversity studies in all aspects of the food and nutrition system is missing in Iran. This study was an effort to analyze food and nutrition security by using NFD, a relatively new metric that takes into account the nutrient composition of food in providing nutrients from farms to markets, and the consumption level. Food purchases from the city play the dominant role in rural household diets. Farm or homestead garden production and village market only play a minor role in rural Zahedan’s diet. Policies that promote diversity through the cultivation of plant and animal species adapted to water shortage and salinity of water and soil are essential in this setting.

More studies are required to clarify whether the NFD score can better reflect diversity compared to other indicators of diversity. Besides, the cut-off points for NFD to rate diversity adequacy should be defined. Additionally, further studies are needed to gain a better understanding of the markets and the effects of their transformations on the nutritional outcomes in the rural area of developing countries. Overall, the NFD score seems to be a promising tool to identify gaps in the diversity of local food and nutrition subsystems and subsequently in planning appropriate interventions and policies, especially in low-income communities.

## Supplementary Information

**Additional file 1.**

## Data Availability

The data sets used and/or analyzed during the current study are available from the authors on reasonable request.

## Abbreviations

NFD: Nutritional Functional Diversity
MAR: Mean Adequacy Ratio
DRI: Dietary Reference Intake
AMEs: Adult Male Equivalent units
NAR: Nutrient Adequacy Ratio
HFIAS: Household Food Insecurity Access Scale
BMI: Body Mass Index
SE: Standard Error
OR: Odds Ratios
CI: Confidence Intervals
SPSS: Statistical Package for the Social Sciences
UCT: Unconditional Cash Transfer
FS: Food secure
FI: Food Insecure
