# Supplementary material for: Is nutritional functional diversity in the rural food and nutrition system associated with food security and nutrient adequacy? A case study of rural areas of Zahedan district, Iran
Source: BMC Public Health. 2022 Apr 14;22:751. doi: 10.1186/s12889-022-13134-8 (PMC9008399; doi:10.1186/s12889-022-13134-8)
Supplement: Supplementary file 1 — Additional file 1. [file 12889_2022_13134_MOESM1_ESM.docx]

Table 1 Association between NFD score of of homestead production with household‎s food security, Zahedan rural areas

| Predictors | Household‎’s food insecurity status† |
| --- | --- |
|  | OR (CI95%) |
| NFD of homestead production | 1.123 (0.930-1.355) |
| Family size | 1.555 (1.178-2.051)^**^ |
| Sex of household‎’s head | 3.370 (0.791-14.360) |
| Education of household‎’s head | 0.751 (0.266-2.116) |
| Employment of household‎’s head | 0.716 (0.228-2.246) |
| Household income | 0.999 (0.998-1.001)^*^ |
| Households received additional subsidy (in addition to the national subsidy)‎ | 0.609 (0.219-1.696) |
| Distance from city | 1.049 (1.022-1.076)^***^ |
| Household‎’s welfare index | 0.468 (0.252-0.869)^*^ |
| Food secure is reference. *p<0.05, **p<0.01, ***p<0.001 | |
| † Controlled for family size, income, households received additional subsidy (in addition to the national subsidy)‎, distance from city, sex and education of household‎’s head, household‎’s welfare index, employment of household‎’s head | |

Table 2 Association between NFD score of household‎’s food processing with household‎s food security, Zahedan rural areas

| Predictors | Household‎’s food insecurity status† |
| --- | --- |
|  | OR (CI95%) |
| NFD of household‎’s food processing ‎ | ‎1.047 (0.899-1.219)‎ |
| Family size | 1.474 (1.149-1.890)^**^ |
| Sex of household‎’s head | 3.033 (0.638-14.422) |
| Education of household‎’s head | 2.159 (0.726-6.421) |
| Employment of household‎’s head | 0.757 (0.179-1.843) |
| Household income | 0.999 (0.998-1.001)^**^ |
| Households received additional subsidy (in addition to the national subsidy)‎ | 0.651 (0.219-1.940) |
| Distance from city | 1.024 (1.005-1.043)^*^ |
| Household‎’s welfare index | 0.795 (0.442-1.431) |
| Food secure is reference. *p<0.05, **p<0.01, ***p<0.001 | |
| † Controlled for family size, income, households received additional subsidy (in addition to the national subsidy)‎, distance from city, sex and education of household‎’s head, household‎’s welfare index, employment of household‎’s head | |

Table 3 Association between NFD score of food purchased from city with household‎s food security, Zahedan rural areas

| Predictors | Household‎’s food insecurity status† |
| --- | --- |
|  | OR (CI95%) |
| NFD of food purchased from city | 0.928 (0.881-0.978)^**^ |
| Family size | 1.581 (1.331-1.878)^***^ |
| Sex of household‎’s head | 2.316 (0.974-5.509) |
| Education of household‎’s head | 1.347 (0.706-2.568) |
| Employment of household‎’s head | 1.126 (0.532-2.386) |
| Household income | 0.999 (0.998-1.001)^***^ |
| Households received additional subsidy (in addition to the national subsidy)‎ | 0.915 (0.449-1.865) |
| Distance from city | 1.010 (0.996-1.023) |
| Household‎’s welfare index | 0.756 (0.534-1.070) |
| Food secure is reference. *p<0.05, **p<0.01, ***p<0.001 | |
| † Controlled for family size, income, households received additional subsidy (in addition to the national subsidy)‎, distance from city, sex and education of household‎’s head, household‎’s welfare index, employment of household‎’s head | |

Table 4 Association between NFD score of food purchased from village with household‎s food security, Zahedan rural areas

| Predictors | Household‎’s food insecurity status† |
| --- | --- |
|  | OR (CI95%) |
| NFD of food purchased from village | 1.051 (1.000-1.104)^*^ |
| Family size | 1.488 (1.244-1.780)^***^ |
| Sex of household‎’s head | 2.961 (1.153-7.604)^*^ |
| Education of household‎’s head | 0.853 (0.433-1.681) |
| Employment of household‎’s head | 1.283 (0.592-2.782) |
| Household income | 0.999 (0.998-1.001)^***^ |
| Households received additional subsidy (in addition to the national subsidy)‎ | 0.736 (0.343-1.578) |
| Distance from city | 1.013 (0.999-1.026) |
| Household‎’s welfare index | 0.617 (0.429-0.888)^**^ |
| Food secure is reference. *p<0.05, **p<0.01, ***p<0.001 | |
| † Controlled for family size, income, households received additional subsidy (in addition to the national subsidy)‎, distance from city, sex and education of household‎’s head, household‎’s welfare index, employment of household‎’s head | |

Table 5 Association between NFD score of consumption (received gift) with household‎s food security, Zahedan rural areas

| Predictors | Household‎’s food insecurity status† |
| --- | --- |
|  | OR (CI95%) |
| NFD of consumption (received gift) ‎ | 1.057 (0.961-1.164) |
| Family size | 1.348 (1.090-1.666) |
| Sex of household‎’s head | 4.482 (1.145-17.548)^*^ |
| Education of household‎’s head | 1.165 (0.499-2.721) |
| Employment of household‎’s head | 1.280 (0.449-3.652) |
| Household income | 0.999 (0.998-1.001) |
| Households received additional subsidy (in addition to the national subsidy)‎ | 0.753 (0.245-2.313) |
| Distance from city | 1.026 (1.008-1.044)^**^ |
| Household‎’s welfare index | 0.464 (0.284-0.758)^**^ |
| Food secure is reference. *p<0.05, **p<0.01, ***p<0.001 | |
| † Controlled for family size, income, households received additional subsidy (in addition to the national subsidy)‎, distance from city, sex and education of household‎’s head, household‎’s welfare index, employment of household‎’s head | |

Table 6 Association between NFD score of native wild ‎vegetables with household‎s food security, Zahedan rural areas

| Predictors | Household‎’s food insecurity status† |
| --- | --- |
|  | OR (CI95%) |
| NFD of native wild vegetables‎ | 1.195 (0.890-1.604) |
| Family size | 1.427 (1.155-1.762)^**^ |
| Sex of household‎’s head | 1.800 (0.605-5.353) |
| Education of household‎’s head | 1.151 (0.507-2.612) |
| Employment of household‎’s head | 0.888 (0.375-2.105) |
| Household income | 0.999 (0.998-1.001)^**^ |
| Households received additional subsidy (in addition to the national subsidy)‎ | 0.958 (0.418-2.195) |
| Distance from city | 1.029 (1.012-1.045)^**^ |
| Household‎’s welfare index | 0.596 (0.385-0.923)^*^ |
| Food secure is reference. *p<0.05, **p<0.01, ***p<0.001 | |
| † Controlled for family size, income, households received additional subsidy (in addition to the national subsidy)‎, distance from city, sex and education of household‎’s head, household‎’s welfare index, employment of household‎’s head | |

Table 7 Association between NFD score of household‎’s diet with household‎s food security, Zahedan rural areas

| Predictors | Household‎’s food insecurity status† |
| --- | --- |
|  | OR (CI95%) |
| NFD of household‎’s diet ‎ | 0.969 (0.895-1.048) |
| Family size | 1.505 (1.281-1.769)^***^ |
| Sex of household‎’s head | 2.203 (0.953-5.092) |
| Education of household‎’s head | 1.140 (0.622-2.090) |
| Employment of household‎’s head | 1.193 (0.584-2.436) |
| Household income | 0.999 (0.998-1.001)^***^ |
| Households received additional subsidy (in addition to the national subsidy)‎ | 0.852 (0.429-1.690) |
| Distance from city | 1.017 (1.005-1.029)^**^ |
| Household‎’s welfare index | 0.703 (0.505-0.979) |
| Food secure is reference. *p<0.05, **p<0.01, ***p<0.001 | |
| † Controlled for family size, income, households received additional subsidy (in addition to the national subsidy)‎, distance from city, sex and education of household‎’s head, household‎’s welfare index, employment of household‎’s head | |

Table 8 Association between NFD score of homestead production with household‎’s Mean Adequacy Ratio, Zahedan rural areas

| Predictors | Household‎’s MAR^1^ ‎ † |
| --- | --- |
|  | B (CI95%) |
| NFD of homestead production ‎ | 0.412 (-0.450-1.275) |
| Family size | -2.106 (-3.211- -1.001)^***^ |
| Age of household‎’s head | -0.058 (-0.227-0.110) |
| Education of household‎’s head | 3.085 (-2.027- 8.198) |
| Employment of household‎’s head | 0.743 (-4.767-6.253) |
| Household income | 2.76 ×10^-6^ (-2.25 ×10^-7^ – 0.6 ×10^-5^) |
| Households received additional subsidy (in addition to the national subsidy)‎ | 1.983 (-3.234- 7.199) |
| Household‎’s welfare index | 3.486 (.648- 6.324)^*^ |
| ^1^Mean Adequacy Ratio, *p<0.05, **p<0.01, ***p<0.001 | |
| † Controlled for family size, age of household‎’s head, household‎’s income, households received additional subsidy (in addition to the national subsidy )‎, education of household‎’s head, household‎’s welfare index, employment of household‎’s head | |

Table 9 Association between NFD score of household‎’s food processing with household‎’s Mean Adequacy Ratio, Zahedan rural areas

| Predictors | Household‎’s MAR^1^ ‎ † |
| --- | --- |
|  | B (CI95%) |
| NFD of household‎’s food processing ‎ | 0.419 (-0.297-1.134) |
| Family size | -2.089 (-3.143- -1.035)^***^ |
| Age of household‎’s head | 0.026 (-0.139-0.191) |
| Education of household‎’s head | 2.920 (-2.034-7.874) |
| Employment of household‎’s head | -1.283 (-6.838 - 4.272) |
| Household income | 3.16 ×10^-6^ (4.00 ×10^-7^ -0.6 ×10^-5^)^*^ |
| Households received additional subsidy (in addition to the national subsidy)‎ | 1.538 (-3.855- 6.930) |
| Household‎’s welfare index | 2.627 (-0.151- 5.405) |
| ^1^Mean Adequacy Ratio, *p<0.05, **p<0.01, ***p<0.001 | |
| † Controlled for family size, age of household‎’s head, household‎’s income, households received additional subsidy (in addition to the national subsidy )‎, education of household‎’s head, household‎’s welfare index, employment of household‎’s head | |

Table 10 Association between NFD score of food purchased from city with household‎’s Mean Adequacy Ratio, Zahedan rural areas

| Predictors | Household‎’s MAR^1^ ‎ † |
| --- | --- |
|  | B (CI95%) |
| NFD of food purchased from city ‎ | 0.355 (0.110 - 0.600)^**^ |
| Family size | -1.468 (-2.197- -0.739)^***^ |
| Age of household‎’s head | 0.034 (-0.086- 0.154) |
| Education of household‎’s head | 1.741 (-1.731 - 5.212) |
| Employment of household‎’s head | 2.552 (-1.319- 6.423) |
| Household income | 1.30×10^-6^ (-6.56×10^-7^-0.41× 10^-5^) |
| Households received additional subsidy (in addition to the national subsidy)‎ | -.773 (-4.697- 3.150) |
| Household‎’s welfare index | 3.920 (2.071- 5.770)^***^ |
| ^1^Mean Adequacy Ratio, *p<0.05, **p<0.01, ***p<0.001 | |
| † Controlled for family size, age of household‎’s head, household‎’s income, households received additional subsidy (in addition to the national subsidy )‎, education of household‎’s head, household‎’s welfare index, employment of household‎’s head | |

Table 11 Association between NFD score of food purchased from village‎ with household‎’s Mean Adequacy Ratio, Zahedan rural areas

| Predictors | Household‎’s MAR^1^ ‎ † |
| --- | --- |
|  | B (CI95%) |
| NFD of food purchased from village ‎ | -0.017 (-0.266-0.232) |
| Family size | -1.616 (-2.400- -0.833)^***^ |
| Age of household‎’s head | 0.052 (-0.086-0.189) |
| Education of household‎’s head | 0.691 (.691-4.403) |
| Employment of household‎’s head | 2.145 (-1.921-6.210) |
| Household income | 1.56 ×10^-6^ (-4.7×10^-7^ – 0.4 ×10^-5^) |
| Households received additional subsidy (in addition to the national subsidy)‎ | -0.575 (-4.769- 3.619) |
| Household‎’s welfare index | 5.378 (3.411-7.345)^***^ |
| ^1^Mean Adequacy Ratio, *p<0.05, **p<0.01, ***p<0.001 | |
| † Controlled for family size, age of household‎’s head, household‎’s income, households received additional subsidy (in addition to the national subsidy )‎, education of household‎’s head, household‎’s welfare index, employment of household‎’s head | |

Table 12 Association between NFD score of consumption (received gift) from village‎ with household‎’s Mean Adequacy Ratio, Zahedan rural areas

| Predictors | Household‎’s MAR^1^ ‎ † |
| --- | --- |
|  | B (CI95%) |
| NFD of consumption (received gift) | 0.458 (-0.059-0.976) |
| Family size | -0.708 |
| Age of household‎’s head | -0.708 (-1.738-0.322) |
| Education of household‎’s head | 0.063 (-4.889-5.015) |
| Employment of household‎’s head | 0.194 (-5.489-5.877) |
| Household income | 2.12 ×10^-6^ (-7.2 ×10^-7^- 0.5 ×10^-5^) |
| Households received additional subsidy (in addition to the national subsidy)‎ | 3.062 (-2.890-9.014) |
| Household‎’s welfare index | 5.561 (2.876-8.245)^***^ |
| ^1^Mean Adequacy Ratio, *p<0.05, **p<0.01, ***p<0.001 | |
| † Controlled for family size, age of household‎’s head, household‎’s income, households received additional subsidy (in addition to the national subsidy )‎, education of household‎’s head, household‎’s welfare index, employment of household‎’s head | |

Table 13 Association between NFD score of native wild vegetables from village‎ with household‎’s Mean Adequacy Ratio, Zahedan rural areas

| Predictors | Household‎’s MAR^1^ ‎ † |
| --- | --- |
|  | B (CI95%) |
| NFD of native wild vegetables | 0.876 (-0.504-2.256) |
| Family size | -2.158 (-3.090--1.225)^***^ |
| Age of household‎’s head | 0.038 (-0.119-.194) |
| Education of household‎’s head | 3.176 (-1.070-7.421) |
| Employment of household‎’s head | 1.930 (-2.615-6.476) |
| Household income | 1.96 ×10^-6^ (-5.4×10^-7^-0.4×10^-5^) |
| Households received additional subsidy (in addition to the national subsidy)‎ | .780 (-3.731- 5.292 ) |
| Household‎’s welfare index | 2.850 (0.540- 5.161)^*^ |
| ^1^Mean Adequacy Ratio, *p<0.05, **p<0.01, ***p<0.001 | |
| † Controlled for family size, age of household‎’s head, household‎’s income, households received additional subsidy (in addition to the national subsidy )‎, education of household‎’s head, household‎’s welfare index, employment of household‎’s head | |

Table 14 Association between NFD score of homestead production with BMI of household‎ head, Zahedan rural areas

| Predictors | BMI¹ of household head † |
| --- | --- |
|  | B (CI95%) |
| NFD of homestead production | 0.069 (-0.298-0.435) |
| Family size | 0.007 (-0.461- 0.475) |
| Age of household‎’s head | -0.007 (-0.076- 0.062) |
| Education of household‎’s head | -0.812 (-2.969-1.346) |
| Employment of household‎’s head | -1.234 (-3.474-1.006) |
| Household income | 1.51×10^-6^ (2.50×10^-7^-0.3×10^-5^) |
| Households received additional subsidy (in addition to the national subsidy)‎ | 0.766 (-1.328- 2.860) |
| Residence status | 0.436 (-2.361-3.234) |
| Household‎’s welfare index | 1.026 (-0.183- 2.236) |
| ¹Body Mass Index, *p<0.05, **p<0.01, ***p<0.001 | |
| † Controlled for family size, age of household‎’s head, household‎’s income, households received additional subsidy (in addition to the national subsidy), residence status, education of household‎’s head, household‎’s welfare index, employment of household‎’s head | |

Table 15 Association between NFD score of household‎’s food processing with BMI of household‎ head, Zahedan rural areas

| Predictors | BMI¹ of household head † |
| --- | --- |
|  | B (CI95%) |
| NFD of household‎’s food processing | 0.033 (-0.238-0.304) |
| Family size | -0.115 (-0.538-0.309) |
| Age of household‎’s head | -0.031 (-0.094-0.032) |
| Education of household‎’s head | -0.011 ) -1.906-1.884) |
| Employment of household‎’s head | -0.313 (-2.402-1.777) |
| Household income | 1.51×10^-6^ (4.5×10^-7^-0.3×10^-5^) |
| Households received additional subsidy (in addition to the national subsidy)‎ | 0.731 (-1.285-2.747) |
| Residence status | 0.073 (-2.164-2.310) |
| Household‎’s welfare index | 0.700 (-0.400-1.800) |
| ¹Body Mass Index, *p<0.05, **p<0.01, ***p<0.001 | |
| † Controlled for family size, age of household‎’s head, household‎’s income, households received additional subsidy (in addition to the national subsidy), residence status, education of household‎’s head, household‎’s welfare index, employment of household‎’s head | |

Table 16 Association between NFD score of food purchased from city with BMI of household‎ head, Zahedan rural areas

| Predictors | BMI¹ of household head † |
| --- | --- |
|  | B (CI95%) |
| NFD of food purchased from city | 0.087 (-0.006-0.180) |
| Family size | 0.344 (0.067-0.621) |
| Age of household‎’s head | -0.011 (-0.056-0.033) |
| Education of household‎’s head | 0.320 (-0.986-1.626) |
| Employment of household‎’s head | -0.854 (-2.302-.594) |
| Household income | 6.58 ×10^-7^ (-6.96×10^-8^- 0.1×10^-5^) |
| Households received additional subsidy (in addition to the national subsidy)‎ | 0.541 (-0.905-1.988) |
| Residence status | -0.486 (-1.848- 0.875) |
| Household‎’s welfare index | 1.053 (0.334-1.773)^**^ |
| ¹Body Mass Index, *p<0.05, **p<0.01, ***p<0.001 | |
| † Controlled for family size, age of household‎’s head, household‎’s income, households received additional subsidy (in addition to the national subsidy), residence status, education of household‎’s head, household‎’s welfare index, employment of household‎’s head | |

Table 17 Association between NFD score of food purchased from village with BMI of household‎ head, Zahedan rural areas

| Predictors | BMI¹ of household head † |
| --- | --- |
|  | B (CI95%) |
| NFD of food purchased from village | -0.034 (-0.125-0.057) |
| Family size | 0.345 (0.053-0.637)^*^ |
| Age of household‎’s head | 0.015 (-0.036-0.065) |
| Education of household‎’s head | 0.571 (-0.793-1.935) |
| Employment of household‎’s head | -0.854 (-2.347-0.640) |
| Household income | 7.27×10^-7^ (-1.40×10^-8^-0.1×10^-5^)^*^ |
| Households received additional subsidy (in addition to the national subsidy)‎ | 0.043 (-1.462-1.549) |
| Residence status | -0.220 (-1.681-1.241) |
| Household‎’s welfare index | 1.382 (0.640-2.124)^***^ |
| ¹Body Mass Index, *p<0.05, **p<0.01, ***p<0.001 | |
| † Controlled for family size, age of household‎’s head, household‎’s income, households received additional subsidy (in addition to the national subsidy), residence status, education of household‎’s head, household‎’s welfare index, employment of household‎’s head | |

Table 18 Association between NFD score of consumption (received gift) with BMI of household‎ head, Zahedan rural areas

| Predictors | BMI¹ of household head † |
| --- | --- |
|  | B (CI95%) |
| NFD of of consumption (received gift)‎ | 0.058 (-0.122-0.238) |
| Family size | 0.582 (0.206-0.959)^**^ |
| Age of household‎’s head | -0.075 (-0.140- -0.010)^*^ |
| Education of household‎’s head | 0.196 (-1.572-1.963) |
| Employment of household‎’s head | 0.025 (-2.001-2.052) |
| Household income | 2.79 ×10^-7^ (-7.35×10^-7^ – 0.1×10^-5^) |
| Households received additional subsidy (in addition to the national subsidy)‎ | 3.477 (1.386-5.568)^**^ |
| Residence status | 0.341 (-1.570-2.253) |
| Household‎’s welfare index | 2.370 (1.362-3.378)^***^ |
| ¹Body Mass Index, *p<0.05, **p<0.01, ***p<0.001 | |
| † Controlled for family size, age of household‎’s head, household‎’s income, households received additional subsidy (in addition to the national subsidy), residence status, education of household‎’s head, household‎’s welfare index, employment of household‎’s head | |

Table 19 Association between NFD score of native wild vegetables with BMI of household‎ head, Zahedan rural areas

| Predictors | BMI¹ of household head † |
| --- | --- |
|  | B (CI95%) |
| NFD of native wild vegetables | 0.078 (-0.468-0.624) |
| Family size | 0.222 (-0.155-0.599) |
| Age of household‎’s head | 0.007 (-0.054-0.068) |
| Education of household‎’s head | 0.039 (-1.651-1.729) |
| Employment of household‎’s head | -0.928 (-2.706-0.849) |
| Household income | 1.03×10^-6^  (3.61×10^-8^-0.2×10^-5^)^P^ |
| Households received additional subsidy (in addition to the national subsidy)‎ | 0.497 (-1.255-2.249) |
| Residence status | 0.213 (-1.833-2.260) |
| Household‎’s welfare index | 1.058 (0.104-2.012)^*^ |
| ¹Body Mass Index, *p<0.05, **p<0.01, ***p<0.001 | |
| † Controlled for family size, age of household‎’s head, household‎’s income, households received additional subsidy (in addition to the national subsidy), residence status, education of household‎’s head, household‎’s welfare index, employment of household‎’s head | |

Table 20 Association between NFD score of household‎’s diet with BMI of household‎ head, Zahedan rural areas

| Predictors | BMI¹ of household head † |
| --- | --- |
|  | B (CI95%) |
| NFD of household‎’s diet | 0.033 (-0.124-0.191) |
| Family size | 0.386 (0.117-0.655)^**^ |
| Age of household‎’s head | -0.014 (-0.058-0.030) |
| Education of household‎’s head | 0.120 (-1.132-1.372) |
| Employment of household‎’s head | -0.970 (-2.377-0.437) |
| Household income | 7.69×10^-7^ (6.38×10^-8^ -0.1×10^-5^)^*^ |
| Households received additional subsidy (in addition to the national subsidy)‎ | 0.646 (-0.756-2.047) |
| Residence status | -0.311 (-1.649-1.027) |
| Household‎’s welfare index | 1.229 (0.521-1.938)^**^ |
| ¹Body Mass Index, *p<0.05, **p<0.01, ***p<0.001 | |
| † Controlled for family size, age of household‎’s head, household‎’s income, households received additional subsidy (in addition to the national subsidy), residence status, education of household‎’s head, household‎’s welfare index, employment of household‎’s head | |

Table 21 Association between NFD score of homestead production with waist circumference of household‎ head, Zahedan rural areas

| Predictors | Waist circumference of household‎ head † |
| --- | --- |
|  | B (CI95%) |
| NFD of homestead production | 0.147 (-0.803-1.098) |
| Family size | 0.860 (-0.416-2.136) |
| Age of household‎’s head | 0.040 (-0.122-0.202) |
| Sex of household‎’s head | 15.676 (-10.663-42.014) |
| Married status of household‎’s head | -15.740 (-40.882 – 9.403) |
| Household income | 2.88 ×10^-6^ (2.16×10^-7^-0.6×10^-5^)^*^ |
| Households received additional subsidy (in addition to the national subsidy)‎ | 0.957 (-4.611-6.525) |
| Household‎’s welfare index | 1.820 (-1.301-4.941) |
| *p<0.05, **p<0.01, ***p<0.001 | |
| †Controlled for family size, age and sex of household‎’s head, household‎’s income, households received additional ‎subsidy (in addition to the national subsidy ), household‎’s welfare index, married status of household‎’s head | |

Table 22 Association between NFD score of household‎’s food processing with waist circumference of household‎ head, Zahedan rural areas

| Predictors | Waist circumference of household‎ head † |
| --- | --- |
|  | B (CI95%) |
| NFD of household‎’s food processing | 0.063 (-0.650-0.777) |
| Family size | 0.432 (-0.706-1.569) |
| Age of household‎’s head | -0.005 (-0.158-0.147) |
| Sex of household‎’s head | 9.074 ( -15.096-33.245) |
| Married status of household‎’s head | -15.058 (-38.007-7.890) |
| Household income | 4.44×10^-6^ (0.2×10^-5^-0.7×10^-5^)^***^ |
| Households received additional subsidy (in addition to the national subsidy)‎ | -1.115 (-6.296-4.065) |
| Household‎’s welfare index | 2.410 (-0.334-5.154) |
| *p<0.05, **p<0.01, ***p<0.001 | |
| †Controlled for family size, age and sex of household‎’s head, household‎’s income, households received additional ‎subsidy (in addition to the national subsidy ), household‎’s welfare index, married status of household‎’s head | |

Table 23 Association between NFD score of food purchased from city with waist circumference of household‎ head, Zahedan rural areas

| Predictors | Waist circumference of household‎ head † |
| --- | --- |
|  | B (CI95%) |
| NFD of food purchased from city | 0.163 (-0.072-0.398) |
| Family size | 1.174 (0.421-1.928)^**^ |
| Age of household‎’s head | 0.038 (-0.068-0.145) |
| Sex of household‎’s head | -0.700 (-12.458-11.059) |
| Married status of household‎’s head | -0.257 (-11.69-11.183) |
| Household income | 2.01 ×10^-6^ (4.79×10^-7^-0.4×10^-5^)* |
| Households received additional subsidy (in addition to the national subsidy)‎ | 0.371 (-3.356-4.098) |
| Household‎’s welfare index | 2.415 (0.671-4.159)^**^ |
| *p<0.05, **p<0.01, ***p<0.001 | |
| †Controlled for family size, age and sex of household‎’s head, household‎’s income, households received additional ‎subsidy (in addition to the national subsidy ), household‎’s welfare index, married status of household‎’s head | |

Table 24 Association between NFD score of food purchased from village with waist circumference of household‎ head, Zahedan rural areas

| Predictors | Waist circumference of household‎ head † |
| --- | --- |
|  | B (CI95%) |
| NFD of food purchased from village | -0.105 (-0.335-0.124) |
| Family size | 1.210 (0.405-2.015)^**^ |
| Age of household‎’s head | 0.086 (-0.036-0.208) |
| Sex of household‎’s head | -2.368 (-14.109-9.372) |
| Married status of household‎’s head | 0.130 (-11.223-11.483) |
| Household income | 2.34× 10^-6^ (7.5×10^-7^ – 0.4×10^-5^)^**^ |
| Households received additional subsidy (in addition to the national subsidy)‎ | -0.415 (-4.368-3.537) |
| Household‎’s welfare index | 3.323 (1.477-5.169)^***^ |
| *p<0.05, **p<0.01, ***p<0.001 | |
| †Controlled for family size, age and sex of household‎’s head, household‎’s income, households received additional ‎subsidy (in addition to the national subsidy ), household‎’s welfare index, married status of household‎’s head | |

Table 25 Association between NFD score of consumption (received gift) with waist circumference of household‎ head, Zahedan rural areas

| Predictors | Waist circumference of household‎ head † |
| --- | --- |
|  | B (CI95%) |
| NFD of consumption (received gift)‎ | 0.345 (-0.136-0.827) |
| Family size | 1.627 (0.559-2.695)^**^ |
| Age of household‎’s head | -0.083 (-0.244-0.079) |
| Sex of household‎’s head | -7.783 (-31.99-16.43) |
| Married status of household‎’s head | 5.861 (-17.98-29.71) |
| Household income | 5.86 ×10^-7^ (-0.2×10^-5^-0.3×10^-5^) |
| Households received additional subsidy (in addition to the national subsidy)‎ | 3.720 (-2.309-9.748) |
| Household‎’s welfare index | 5.392 (2.833-7.952)^***^ |
| *p<0.05, **p<0.01, ***p<0.001 | |
| †Controlled for family size, age and sex of household‎’s head, household‎’s income, households received additional ‎subsidy (in addition to the national subsidy ), household‎’s welfare index, married status of household‎’s head | |

Table 26 Association between NFD score of native wild vegetables with waist circumference of household‎ head, Zahedan rural areas

| Predictors | Waist circumference of household‎ head † |
| --- | --- |
|  | B (CI95%) |
| NFD of native wild vegetables | 0.217 (-1.113-1.546) |
| Family size | 1.076 (0.100-2.053)^*^ |
| Age of household‎’s head | 0.071 (-0.069-0.211) |
| Sex of household‎’s head | 5.726 (-11.272-22.725) |
| Married status of household‎’s head | -5.922 (-22.181-10.337) |
| Household income | 2.65 ×10^-6^ (5.8×10^-7^-0.5×10^-5^)^*^ |
| Households received additional subsidy (in addition to the national subsidy)‎ | -0.926 (-5.358-3.505) |
| Household‎’s welfare index | 2.275 (0.070-4.479)^*^ |
| *p<0.05, **p<0.01, ***p<0.001 | |
| †Controlled for family size, age and sex of household‎’s head, household‎’s income, households received additional ‎subsidy (in addition to the national subsidy ), household‎’s welfare index, married status of household‎’s head | |

Table 27 Association between NFD score of household‎’s diet with waist circumference of household‎ head, Zahedan rural areas

| Predictors | Waist circumference of household‎ head † |
| --- | --- |
|  | B (CI95%) |
| NFD of household‎’s diet | 0.130 (-0.268-0.528) |
| Family size | 0.130 (-0.268-0.528)^***^ |
| Age of household‎’s head | 1.317 (0.587-2.048) |
| Sex of household‎’s head | 0.035 (-0.069-0.139) |
| Married status of household‎’s head | 0.061 (-11.279-11.400) |
| Household income | 2.13×10^-6^ (6.33×10^-7^-0.4×10^-5^)^**^ |
| Households received additional subsidy (in addition to the national subsidy)‎ | 0.434 (-3.206-4.075) |
| Household‎’s welfare index | 2.536 (0.808-4.264)^**^ |
| *p<0.05, **p<0.01, ***p<0.001 | |
| †Controlled for family size, age and sex of household‎’s head, household‎’s income, households received additional ‎subsidy (in addition to the national subsidy ), household‎’s welfare index, married status of household‎’s head | |
